# Patient-derived organoids in human cancer: a platform for fundamental research and precision medicine

**DOI:** 10.1186/s43556-023-00165-9

**Published:** 2024-02-12

**Authors:** Shanqiang Qu, Rongyang Xu, Guozhong Yi, Zhiyong Li, Huayang Zhang, Songtao Qi, Guanglong Huang

**Affiliations:** 1 Department of Neurosurgery, Nanfang Hospital, Southern Medical University, Guangzhou Dadao Bei Street 1838, Guangzhou, 510515 Guangdong China; 2 The Laboratory for Precision Neurosurgery, Nanfang Hospital, Southern Medical University, Guangzhou, 510515 Guangdong China; 3 Nanfang Glioma Center, Guangzhou, 510515 Guangdong China; 4 Institute of Brain disease, Nanfang Hospital, Southern Medical University, Guangzhou Dadao Bei Street 1838, Guangzhou, 510515 Guangdong China; 5 The First Clinical Medical College of Southern Medical University, Guangzhou, 510515 Guangdong China

**Keywords:** Organoids, Cancer, Emerging model, Preclinical models, Precision medicine

## Abstract

Cancer is associated with a high degree of heterogeneity, encompassing both inter- and intra-tumor heterogeneity, along with considerable variability in clinical response to common treatments across patients. Conventional models for tumor research, such as in vitro cell cultures and in vivo animal models, demonstrate significant limitations that fall short of satisfying the research requisites. Patient-derived tumor organoids, which recapitulate the structures, specific functions, molecular characteristics, genomics alterations and expression profiles of primary tumors. They have been efficaciously implemented in illness portrayal, mechanism exploration, high-throughput drug screening and assessment, discovery of innovative therapeutic targets and potential compounds, and customized treatment regimen for cancer patients. In contrast to conventional models, tumor organoids offer an intuitive, dependable, and efficient in vitro research model by conserving the phenotypic, genetic diversity, and mutational attributes of the originating tumor. Nevertheless, the organoid technology also confronts the bottlenecks and challenges, such as how to comprehensively reflect intra-tumor heterogeneity, tumor microenvironment, tumor angiogenesis, reduce research costs, and establish standardized construction processes while retaining reliability. This review extensively examines the use of tumor organoid techniques in fundamental research and precision medicine. It emphasizes the importance of patient-derived tumor organoid biobanks for drug development, screening, safety evaluation, and personalized medicine. Additionally, it evaluates the application of organoid technology as an experimental tumor model to better understand the molecular mechanisms of tumor. The intent of this review is to explicate the significance of tumor organoids in cancer research and to present new avenues for the future of tumor research.

## Introduction

Cancer, a perilous affliction in China and worldwide, is unmistakably characterized by an escalating trend in morbidity and mortality year after year. Based on the most recent data unveiled by the International Agency for Research on Cancer (IARC), as documented in the Global Cancer Observatory (GLOBOCAN), there is a forecasted surge of 50% in the prevalence of cancer, resulting in a projected estimate of approximately 30 million cancer cases by the year 2040 [1]. As per the latest reports, the current five-year relative survival rate for malignant tumors in China stands at approximately 40.5%. In comparison to a decade ago, the overall survival rate for malignant tumors in China has witnessed an increase of around 10% points. However, despite this improvement, the incidence and mortality rates of malignant tumors in China continue to rise persistently [2]. There is limited evidence for the use of adjuvant chemoradiotherapy for solid cancer to date [3]. Therefore, the global cancer statue remains a major issue worldwide, researching and developing new effective drugs to treat cancer is extremely urgent. In 2016, Tufts reported a staggering fact that out of the new cancer drugs that entered clinical trials based on results from traditional models, a whopping 92% failed to receive approval in the end [4]. Despite the development of numerous targeted drugs, the majority of them have not been able to attain clinical success. One noteworthy obstacle in this regard lies in the evaluation models employed for this purpose. Approximately 70% of the clinical trials that fail can be linked to the traditional cell and animal models [4, 5]. Presently, the dearth of appropriate in vitro models that can accurately reflect the intricacies of the parental cancer remain a significant research obstacle in the fields of cancer treatment.

As we all known, preclinical tumor models occupy a pivotal position in facilitating mechanistic research and the assessment of innovative therapeutic agents, thus underscoring their indispensable role in scientific exploration. In recent decades, clinical trials have witnessed a remarkable surge in the occurrence of failures concerning novel therapies, despite extensive endeavors focused on target validation and drug optimization, primarily based on conventional traditional preclinical models such as cell-line or patient-derived xenograft (PDX) models [6], as well as murine or nonmurine animal models [7–10]. Conventional cancer models frequently prove inadequate in faithfully replicating parental tumors, leading to the utilization of numerous anticancer drugs that are hampered by limitations including poor drug solubility or stability, suboptimal pharmacokinetic profiles, lack of tumor-specificity, and the manifestation of serious side-effects [11–16]. Previous studies revealed that two-dimensional (2D) cell-line demonstrate their limitations in adequately simulating critical aspects, such as the immune-microenvironment and stromal compartments [17–19]. Furthermore, there are additional constraints to consider, including the loss of genetic heterogeneity in cancer cell-lines due to multiple passages from the parental tumors [20], the occurrence of mouse-specific tumor adaptations in PDX models, and the significant consumption of financial, temporal, and resource-based investments [21, 22].

Organoid technology has emerged as a prominent and autonomous research tool [23–35]. Organoids, formed as intricate three-dimensional (3D) structures, hold the capacity to originate from a wide array of cellular sources, ranging from embryonic stem cells (ESCs) and induced pluripotent stem cells (iPSCs) to somatic stem cells and even cancer cells [36–39]. These 3D tissues, fabricated on a small scale within laboratory settings, closely resemble the native organs in terms of their structure and functionality. Serving as a powerful bridge between conventional in vitro models and in vivo models, this technology exhibits immense potential for clinical applications, particularly in the field of cancer [40]. Tumor modeling stands as a crucial facet within the realm of organoid technology [41–49], encompassing the intricate representation of infection-cancer development [50, 51], mutation-driven tumorigenesis processes [51, 52] and genetic carcinoma [53, 54]. Beyond its pivotal role in cancer modeling, organoid technology exhibits immense potential across various domains, including the assessment of drug efficacy and toxicity [55], advancements in regenerative medicine [56, 57], and the advancement of precision treatment approaches [58, 59]. Only in the recent past have researchers achieved remarkable success in establishing organoids for a wide range of cancer types [60–63].

Patient-derived organoids (PDOs) are miniature 3D models of tumor cells cultivated in the laboratory from primary tumors obtained from patients. PDOs play a crucial role in both fundamental research and precision medicine due to their ability to recapitulate the complex structure and function of human organs. These miniaturized organ models are generated from patient samples and faithfully reproduce the genetic, molecular, and cellular characteristics of the original organ. This makes them invaluable for investigating disease mechanisms, testing drug responses, and developing personalized treatment strategies. By using PDOs, researchers can explore the diverse range of genetic and phenotypic variations observed in individuals, providing crucial insights into disease processes and potential therapeutic targets. PDOs have also emerged as invaluable diagnostic tools in the realm of precision medicine. These organoids, cultivated from patient samples, serve as excellent platforms for in vitro screening of drug responses prior to treatment. The ultimate objective is to furnish healthcare professionals with critical insights that can guide the care of cancer and cystic fibrosis patients, while also facilitating the prediction of treatment outcomes. Thanks to the ceaseless advancements in organoid culture systems and associated experimental techniques, the application of organoids has expanded across diverse research domains.

This review outlines a brief history of organoids and delves into the utilization of tumor organoid techniques within both fundamental research and precision medicine. We emphasize the significance of patient-derived tumor organoid biobanks in facilitating drug development, screening, safety evaluation, and personalized medicine. Additionally, we critically assess the application of organoid technology as an experimental tumor model to unravel the intricate molecular mechanisms underlying tumor biology. Finally, we discuss the limitations and explore the potential of PDOs in tumor research.

## The developmental timeline of organoids

**Fig. 1:**
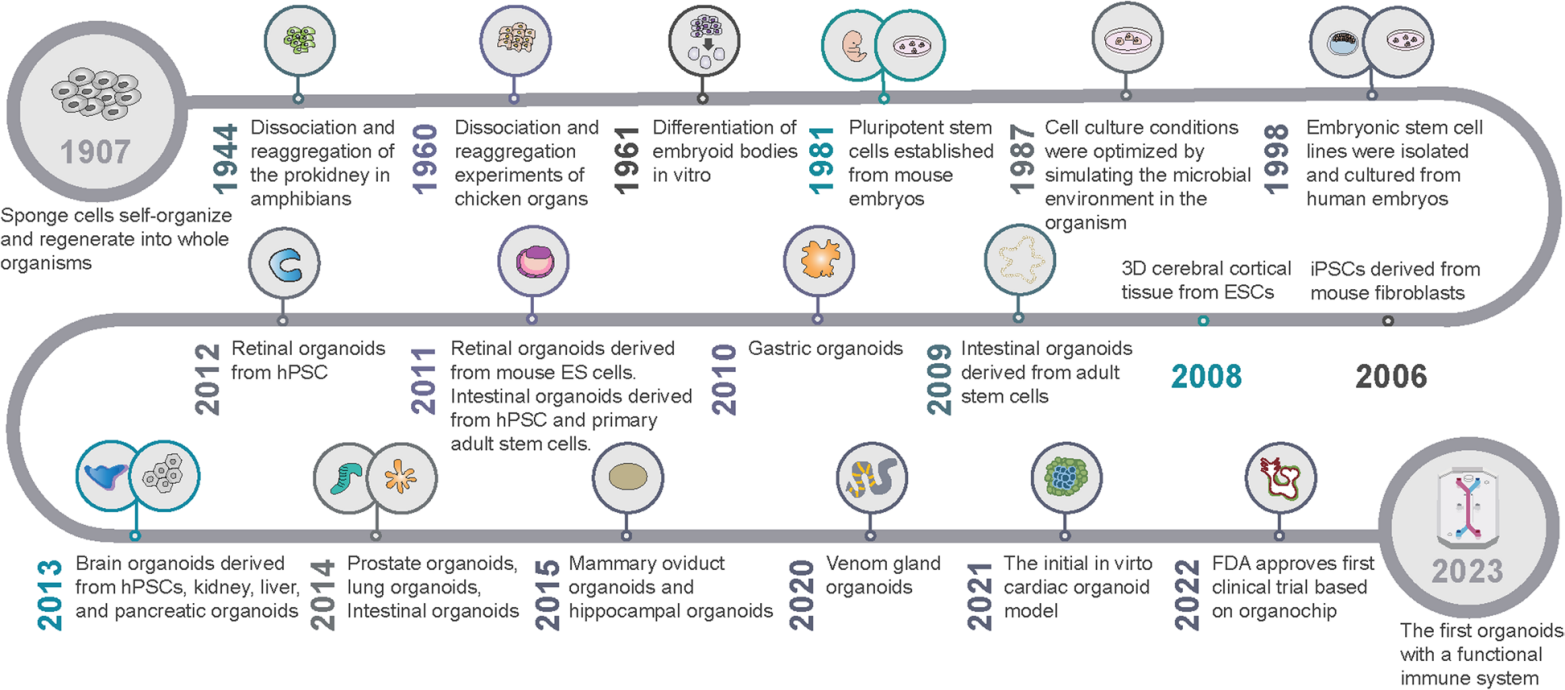
Developmental history of organoids

The term “organoids” encompasses 3D tissue models possessing unique spatial structures that are generated via the in vitro cultivation of pluripotent stem cells or adult stem cells [64]. Termed as “mini-organs,” these structures emulate essential multicellular, anatomical, and even functional attributes of authentic organs [65]. Remarkably, the term “organoid” dates back to 1907 when Henry Van Peters Wilson, a researcher associated with the University of North Carolina at Chapel Hill, hypothesized that individual silica sponge cells, when dissociated from a larger organism, can inherently regenerate into self-organizing, fully functional organisms (Fig. 1) [66]. It was proposed that complex organs/tissue structures could be recreated in vitro. It was thought that this self-organizing and regenerating potential of cells in culture could probably be utilized to recapitulate parts of complete tissues or organs. The popularity of 3D organoid models has soared within the biomedical research community due to their potential use in exploring disease pathogenesis, developing drugs and personalized medicine. The remarkable embryonic tissue plasticity was first hinted by the observations of cell separation and reaggregation in vertebrates by Holtfreter and Weiss in 1944 and 1960, respectively [67, 68]. Several attempts were made to model in vivo organ systems during the 1980s and-90s using dissociation-reaggregation experiments: for example, cells isolated from chick embryos or mouse fetal lungs were dissociated and allowed to reaggregate in culture. Since Martin’s breakthrough discovery in 1981 of isolating embryonic stem cells from mouse embryos, extensive advancements have been made in stem cell research, making them a key area of investigation because of their regenerative capabilities and their immense potential in treating various diseases [69]. Optimizing cell culture conditions, in 1987, allowed Li Maolin and his team to successfully assemble mammary epithelial cells into 3D spheroids and ducts [70]. A significant milestone was achieved in 1998 when Thomson et al. successfully extracted human pluripotent stem cells (hPSCs) from human blastocysts, offering novel prospects in the fields of developmental biology and regenerative medicine [71]. The year 2006 marked a groundbreaking achievement with the reprogramming of mouse and human fibroblasts that led to the successful generation of iPSCs. Kazutoshi Takahashi and Shinya Yamanaka genetically modified mouse fibroblasts to develop murine pluripotent stem cells (PSCs). Induced or embryonic PSCs (iPSCs or ESCs) have effective self-renewal and self-organizing capacities that are lacking in primary cells isolated from tissues or organs. PSCs are organ progenitors that differentiate in culture to give rise to multiple cell types that self-organize to form organ-like complex structures in culture, closely resembling the process of organ formation in embryos until birth, which has significantly impacted the development of 3D organoid models [72]. Thereafter, the establishment of iPSCs in 2007 revolutionized organoid technology to facilitate the development of organoids from single individuals that could be used for diverse in vitro and preclinical studies [73]. The year 2009 marked a groundbreaking discovery in intestinal stem cell research, as Hans Clevers and his team demonstrated the remarkable self-organizing ability of individual Lgr5^+^ intestinal stem cells that were capable of generating organoids composed of various intestinal cell types, such as crypt-villus structures [74]. The year 2009 initiated a new era of cutting-edge organoid technology that delivered a powerful tool for exploring vital biological processes, diagnosing illnesses, and generating personalized therapies. The year 2010 witnessed the successful isolation and recombination of mouse embryonic kidney stem cells that led to the formation of kidney organoids, making way for the development of even more intricate and biologically significant organoid systems [75]. With the advent of in vitro differentiation, researchers were able to leverage the potential of iPSCs to create numerous types of organoids, such as the intestinal and brain organoid. A remarkable development in this context was the derivation of human brain organoids from iPSCs obtained from microcephaly patients in 2013 [43]. Additionally, Lee et al. discovered that a 3D co-cultivation approach of endothelial cells and adult bronchiolar alveolar stem cells had the ability to generate pulmonary organoids, marking yet another advancement in the field of organoid technology [76]. The year 2015 experienced the advent of mammary gland, fallopian tube, and hippocampal organoids which marked a significant leap in the field of organoid technology. Furthermore, a recent study in 2020 accomplished the production of snake venom gland organoids, expanding the scope of organoid research [77]. The year 2020 witnessed a remarkable achievement in the realm of organoid technology as Hongjun Song and his colleagues established the glioblastoma multiforme (GBM) organoid model that retained numerous vital traits of GBM [78]. This model could be effectively leveraged to investigate personalized treatment strategies for patients. In May 2021, the Institute of Molecular Biotechnology of the Austrian Academy of Sciences announced the development of the world’s first externally self-organizing heart organoid model, as reported by their research team [79]. They have successfully cultured a heart organoid model using human pluripotent stem cells, which has the remarkable ability to autonomously generate cavities and exhibit pulsations without requiring scaffold support. This advanced model not only allows for the investigation of heart injury repair mechanisms but also serves as a crucial research tool in the study of human cardiogenesis and heart disease mechanisms [79]. In August 2022, the FDA granted approval for the world’s first new drug to enter clinical trials solely based on pre-clinical data obtained from “organochip” research [80]. This milestone marks the first instance where “organochip” experiments have successfully substituted traditional animal experiments. In February 2023, scientists from Cincinnati Children’s Hospital, along with researchers from other institutions, constructed an enhanced model aimed at studying human gastrointestinal diseases [81]. With their persistent endeavors, they have triumphantly pioneered a novel generation of intricate intestinal organoids that effectively encompass crucial components of the functional immune system [81]. Thus far, this represents the initial organoid developed by scientists that includes a fully functional immune system.

Organoids have garnered significant attention and interest in the fields of life sciences and medical research due to their unique application value and broad market potential. While not entirely equivalent to genuine human organs, organoids have successfully replicated the structure and function of real tissues. Furthermore, organoids have demonstrated collaborative potential with technologies such as printing, organ chips, gene editing, and microfluidics, thereby assuming a critical role in tumor modeling, drug development, screening, and precision therapy.

## Organoid generation from tumor tissue

**Fig. 2:**
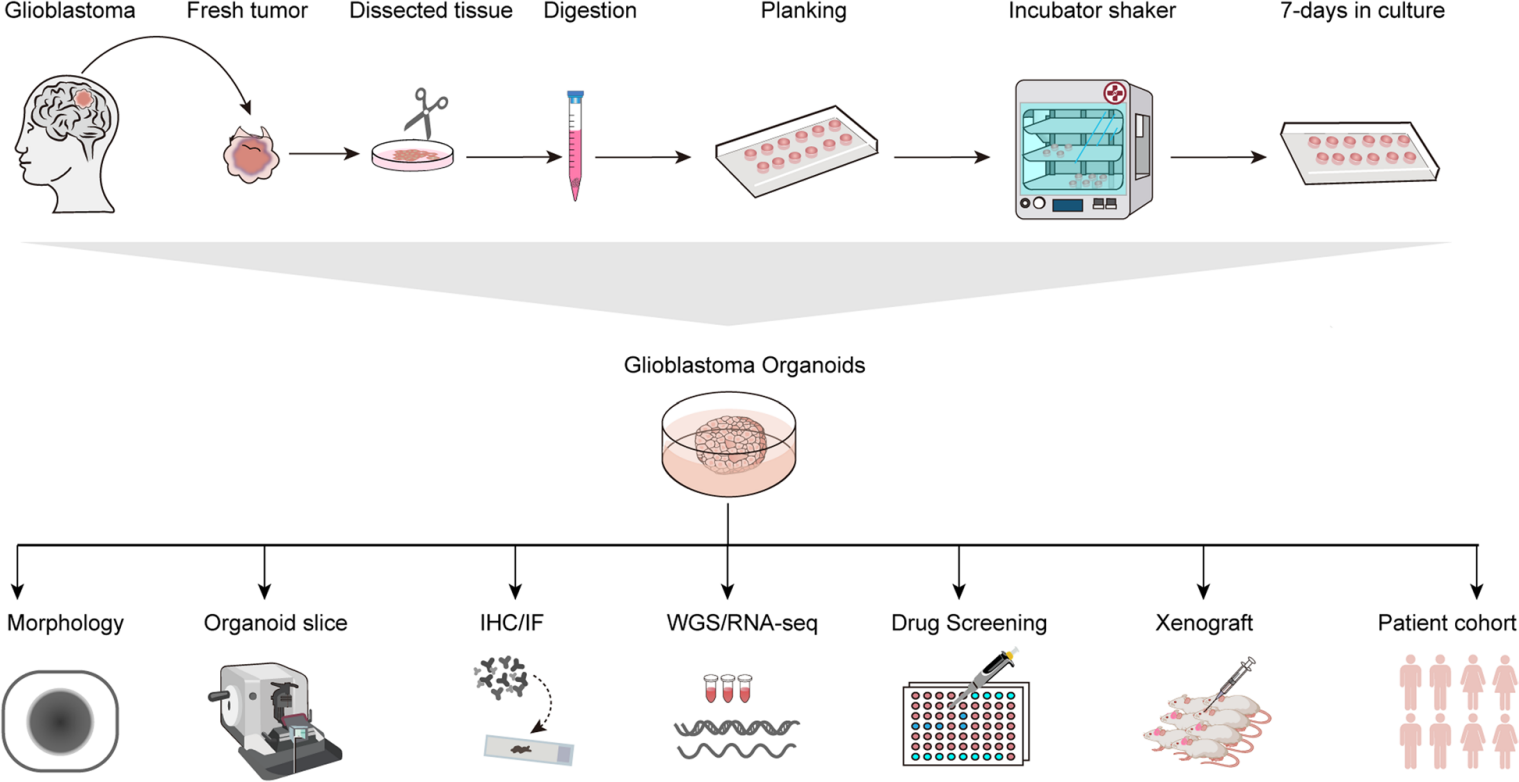
The GBM organoids culture process and subsequent analysis are illustrated schematically. A rapid and dependable technique has been described to create GBM organoids, confirmed by morphology and further verified with immunohistochemistry and immunofluorescence experiments. These organoids have promising applications to functional assays, including WGS and RAN-seq analyses, drug screening, derivation of orthotopic xenografts, and individualized treatment

Organoids are produced by cultivating adult stem cells with specific spatial arrangements in vitro. Currently, there is no universally standardized procedure for constructing organoids, and differences arise in the culture methods of various solid tumor organoids, particularly regarding the culture medium with some growth factors and inhibitors (Table 1). The utilization of diverse combinations of growth factors and inhibitors within the culture medium plays a pivotal role in generating distinct lineages of components within organoids [82]. Whilst the processes employed to generate distinct PDOs may differ to some extent, they typically encompass a core set of fundamental steps that are commonly shared. In this review, we outline the primary operational steps for culturing and identifying PDOs generated from surgically resected GBM tissue [83] (Fig. 2).

- After surgery, GBM tissues were quickly placed in tissue-preservation solution containing 1% penicillin-streptomycin. Tissues were transported to the laboratory, kept at 4℃, and processed within 2 h.
- The tissues were transferred into a super clean bench. The tissues were washed three times with 0.9% sodium chloride (NaCl) solution.
- After washout, the tissues were plated in 60 mm dishes. Minced with scissors into 1–2 mm^3^ pieces.
- For the tryptic digestion of the pieces, digestion solution (6 ml) containing collagenases, DNA zymography and hyaluronic acid was added to tissue pieces. The suspension was placed in a constant temperature shaker at 37℃ and digested at 100 rpm/min for 30 min.
- Digestion was stopped by adding an excess of HBSS buffer, after which the pieces were washed twice with HBSS.
- The cell suspension after digestion was filtered through a 100-mesh sieve to remove undigested tissues. The cell suspensions were collected into a 50 ml centrifuge tube and centrifuged down at 1500 rpm for 3 min.
- After the supernatant had been discarded, the cells were resuspended in 5 ml of red blood cell (RBC) lysing buffer and incubated for 5 min at 37℃, and centrifuged at 1500 rpm for 3 min. The cells were washed three times with 0.9% NaCl solution.
- The supernatant was discarded and fresh glioma organoid medium was added. The cell suspension is added in ultra-low adherent six-well plates and incubated on a rotary shaker (120 rpm) at 37℃. Organoid medium was replaced three times per week, and organoids were maintained for 7–14 days. Photographs were taken daily by microscope.
- Organoids successfully cultivated during passage 3 were regarded as success in organoid formation. Other authentication of organoids, such as whole-genome sequencing (WGS) /RNA-seq, was performed except for morphology, *H&E* staining and immunofluorescence. The research could passage the organoids to expand them and have enough organoid for subsequent experiments.

**Table 1:** Growth factors and small molecule inhibitors applied in organoid cultures

Category	Name	Biological function
Growth factors	EGF	EGF induces proliferative changes by binding to EGF receptors. EGF was also used in the culture of the first intestinal organoids
	Wnt3a	A master regulator orchestrating the regulation of cellular development, proliferation, differentiation, adhesion, and polarity through the activation of Wnt signaling.
	FGF10	The FGF10/FGF receptor 2IIIb axis plays a critical role in organ development. Additionally, FGF10 stimulates the migration and invasion of pancreatic cancer cells and contributes to the development of breast cancer.
	VEGF	VEGF plays a vital role in promoting the expansion of organoids and maintaining the balance of the internal environment.
	FGF7	The signaling of FGF7/FGF receptor 2 actively promotes the growth, invasion, and migration of cancer cells.
	HGF	HGF/Met signaling plays a crucial role in promoting oncogenesis, angiogenesis, and invasion of cancer cells.
	Noggin	Noggin is an endogenous inhibitor of bone morphogenetic protein (BMP), which can regulate cell differentiation, proliferation and apoptosis. Noggin promotes organoid expansion by inhibiting BMP signals.
	R-spondin-1	R-spondin-1 is a ligand for Lgr5, and Lgr5-positive stem cells have been shown to have the potential to self-renew and expand into organoids. R-spondin-1 facilitates the proliferation, division and metastasis of cancer cells.
	Gastrin	Gastrin stimulates tumor growth by enhancing the proliferation of cancer cells and suppressing their apoptosis.
	Nicotinamide	Vitamin PP is an essential nutrient necessary for the long-term cultivation of organoids.
	Neuregulin 1	Neuregulin 1 is a member of the epidermal growth factor family of receptor tyrosine kinase protein ligands (ERBB2, ERBB3, ERBB4), and its activity includes the activation of proliferation, survival, and differentiation of cells.
	Prostaglandin E2	Prostaglandin E2, through the upregulation of vascular endothelial growth factor, facilitates angiogenesis in gastric cancer.
Small molecule inhibitors	CHIR99021	By acting as a small molecule inhibitor of GSK3 signaling, this compound promotes proliferation by inducing the stabilization of β-catenin and c-Myc proteins.
	Y-27,632	Y27632, an inhibitor of Rho kinase, effectively reduces the phenomenon of anoikis in dissociated stem cells. Its inclusion in culture media improves conditions and enhances the long-term in vitro proliferation of tumor epithelial cells.
	A83-01	As a potent inhibitor of transforming growth factor-beta, this compound effectively suppresses the proliferation of organoids.
	SB202190	SB202190, a p38 inhibitor, exerts suppressive effects on the proliferation and migration of cancer cells.

### Cancer PDOs effectively recapitulate the histopathologic characterization of parental tumors

Whether tumor organoids retain the biological characteristics of parental tumors is the current research hotspot. Ruoshi Shi et al. [84] collected 65 lung cancer tissue samples from 2015 to 2017 for organoid construction, of which 57 cases (88%) successfully constructed lung cancer organoids. Organoids and parental tumor markers TTF-1 and TP63 were evaluated by H&E and IHC. The results showed that the organoids reflected the expression patterns of TTF-1 and TP63 in their parent tumors. Yunuo Mao et al. [85] eight human colorectal cancer (CRC) organoids that included tumor types with and without KRAS mutations. They observed that the morphology of the tumor tissue varied from patient to patient, and the corresponding organoids represented the heterogeneous morphology of the tumor tissue. To determine whether organoids used to treat tumors with drugs also have this property, Ping Chen et al. [86] achieved the successful establishment of a comprehensive repository comprising 99 human breast cancer organoids, encompassing primary tumors, drug-resistant variants, and metastatic forms. These organoids were derived from 132 breast cancer tissue samples obtained from 125 patients, yielding an impressive overall success rate of 75%. Morphological and histopathological features of both the organoids and the parental tumors were examined through techniques such as H&E staining and immunohistochemistry for comparative analysis. The findings from the study indicate that breast cancer PDOs faithfully inherit histological attributes from their parental tumors, encompassing growth patterns along with cellular and nuclear atypia, independent of prior drug therapy. Immunohistochemical staining further revealed that the expression patterns of breast cancer markers remained well-preserved within both drug-treated and untreated organoids derived from tumors. Furthermore, in the research conducted by Shaobo Mo et al. [87], a total of 72 surgical tissue samples were acquired, comprising 36 primary CRC tissues and 36 matched colorectal cancer liver metastases. With these samples, the team successfully cultured 58 organoids, including 31 CRC organoids and 27 colorectal cancer liver metastases. Analysis using H&E staining revealed that both colorectal cancer and colorectal cancer liver metastases exhibited certain similarities, such as the presence of thin-walled cystic structures. However, they also retained their distinctive heterogeneity, demonstrating discernible variations between the two. Subsequently, crucial molecular markers, such as Ki67, CDX2, β-catenin, CK-pan, and CK20 expression, were assessed utilizing immunohistochemistry (IHC). PDOs in liver metastases of colorectal cancer preserves the histopathological structure of the parent tumor, thereby preserving homology with the source individual and heterogeneity among individuals.

### Genetic characterization of tumor organoids

To evaluate the genomic consistency between organoids and parental tumor tissues, Ruoshi Shi et al. [84] compared the somatic mutational spectrum and replicative number variation between 9 groups of lung cancer organoids and their parent tumors using WES. The findings demonstrated a remarkably high degree of concordance in the mutant spectrum between organoids and parental tumors, suggesting that the culture conditions employed did not compromise the inherent stability of the tumor genome. Moreover, analysis of copy number variation (CNV) revealed that the CNV profiles observed in the parental tumors were largely maintained throughout the course of organoid cultivation. Simultaneously, to ascertain the preservation of the gene expression profile from the parent tumor tissue in the organoids, RNA-seq analysis was conducted. The results revealed an overall gene expression correlation of 0.59 between the patient’s tumor and the organoids, whereas the correlation between PDX and organoids was 0.8, indicating a slightly higher resemblance. Yunuo Mao et al. [85] used single nucleotide variant (SNV) and whole exome sequencing (WES) to understand tumor-specific organoid gene mutations. The study revealed the presence of identical driver gene mutations in both the organoids and parental tumor. These mutations were categorized into various types based on their involvement in signaling pathways or associated genes, including the Wnt/β-catenin, RAS/MAPK, PI3K/AKT, TGF, and TP53-related pathways. These key driver genes, APC and TP53, which are frequently associated with CRC, were consistently detected across all eight organoid lines established in this study. In terms of the transcriptome, organoids exhibit a preserved expression of significant tumor-related signaling pathways, notably the PI3K/AKT, Wnt/β-catenin, and TP53 pathways. To determine whether drug-derived and therapeutic tumor organoids retain parental genomic signatures, Ping Chen et al. [86] performed WES paired sequencing on 12 breast cancer organoids and parental tumor tissues, including primary, drug-resistant, and metastatic breast cancers. Genome-wide exome analysis of cDNA showed that DNA copy number loss and increase in parental tumors are typically retained across the genome in breast cancer organoids, including those produced by drug therapy and metastatic tumors. On average, 63.8% of CNV genes identified in primary tumors were observed to be retained in organoids. Subsequently, a comparison was made between SNV genes in parental tumors and organoids. On average, a remarkable 82.1% of the selected cancer-related genes detected in primary tumors were successfully maintained within the organoids. Shanbo Mo et al. [87] reported that colorectal cancer liver metastasis organoids retained 88.9% of CRC gene mutations from the corresponding tumor (88.5% for CRC organoids and 89.2% for colorectal cancer liver metastasis organoids).

The development of GBM involves various genetic changes, such as *EGFR* amplification, *IDH1/2* mutation, *TERT* mutation, *PTEN* mutation, *NF1* mutation, *PDGFRα* alterations, *TP53* mutation, *RB* alterations, *CDKN2A* deletion, and *MGMT* promoter methylation [88, 89]. Fadi Jacob and colleagues reported that GBM organoids exhibit gene expression characteristics and genomic landscapes that are similar to their corresponding parental tumors by conducting extensive RNA sequencing on 12 patients [78]. Comparisons of the transcriptome between GBM organoids and their corresponding parental tumors demonstrate a high degree of similarity. Exome sequencing also reveals similar frequencies of somatic mutation and CNV between the two. These findings suggest that GBM organoids largely preserve the molecular characteristics of tumor tissue and the heterogeneity of tumor cells.

In summary, organoids do exhibit a degree of preservation of the genomic features found in their parental tumors, albeit with certain distinctions between them. Generally, a higher number of CAN and SNV genes were observed in the organoids compared to the parental tumors.

## Comparison of the preclinical tumor models

**Fig. 3:**
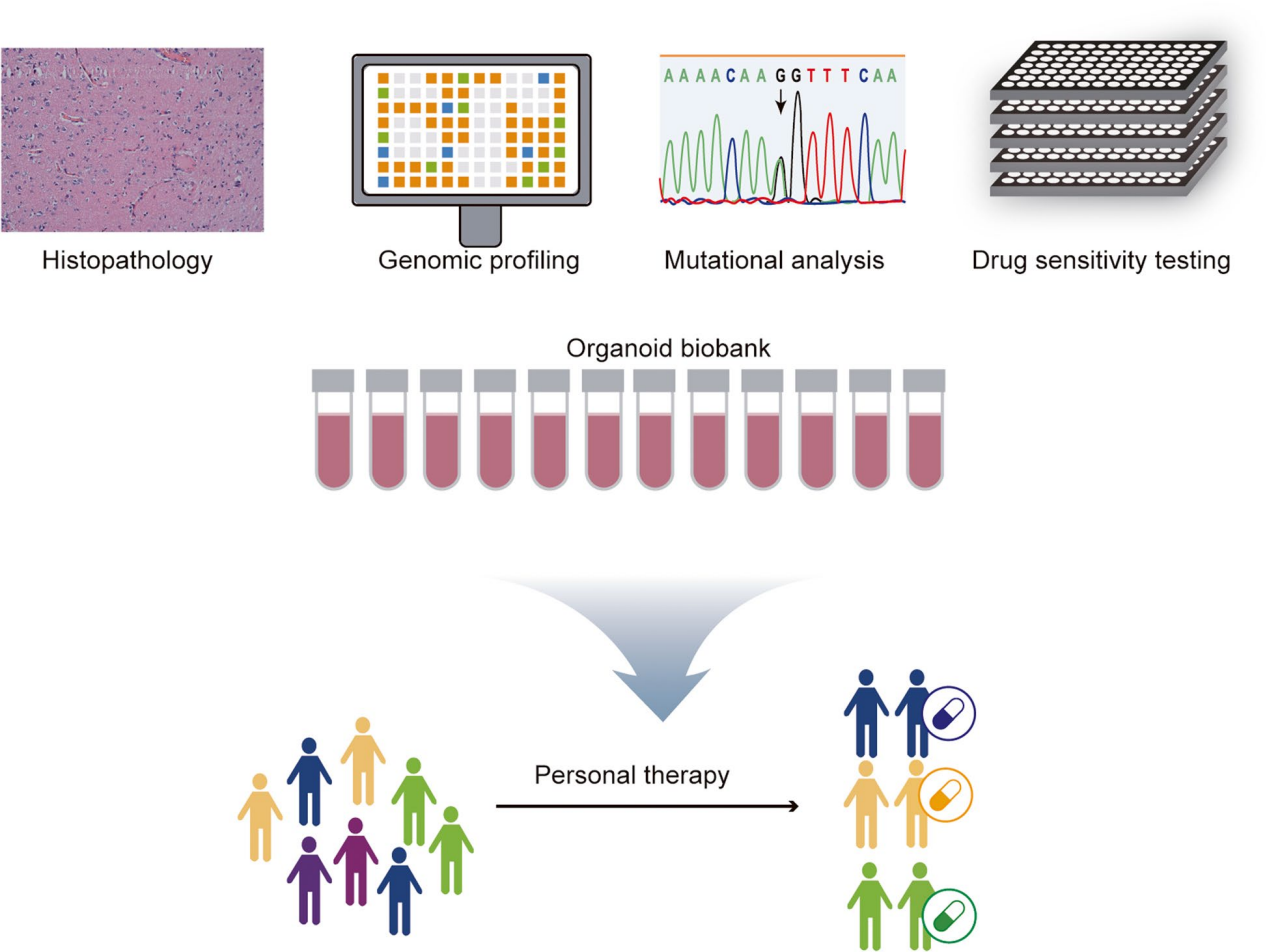
Tumor organoids display histopathological characteristics, genetic profiles, mutational landscape, and even treatment responses that closely resemble the parental tissues

Cancer, characterized by its elevated clinical mortality rates, poses significant challenges in research and drug development. Presently, cancer investigations largely rely on cell and animal models. Regrettably, these traditional preclinical models encounter limitations in truly replicating the intricate physiological structure and functionality of humans. Consequently, they impede progress in cancer research and drug development. For instance, twenty-five years ago, Mina Bissell observed an intriguing phenomenon: mouse mammary cells cultured in a petri dish failed to secrete milk [90]. However, when these mammary cells were cultured in an environment that mimicked the conditions of the cells in the body, they formed 3D structures and resumed milk production [90, 91]. This phenomenon indicates that the behavior of cells is heavily influenced by their extracellular environment, and that cells cultured in a 2D environment lose their tissue structure. The limitations of the 2D culture method are becoming increasingly apparent (Table 2). Firstly, the cell culture system is devoid of immune cell functionality, microenvironment, interstitial components, and organ specificity [92–94]. Secondly, the extended growth of cells in a 2D environment of a petri dish may lead to the selection of a dominant clone pair. Thirdly, there are also notable distinctions between the traditional 2D model and the 3D model in the tissue microenvironment, including extracellular matrix (ECM) and cell polarity [94]. The unrestricted access to nutrients in these models inaccurately replicates the metabolic environment in vivo. Scientists frequently resort to immortal cell lines due to their convenience in acquisition as well as their capacity to sustain prolonged survival and proliferation under laboratory conditions, which are comparatively less demanding in terms of procurement and upkeep. However, it is worth noting that the physiological structure and cellular behavior of these cell lines, which have attained immortality through genetic manipulation, diverge significantly from their original human cellular counterparts. As a consequence, the comprehension of diseases and the assessment of drug effects may exhibit substantial disparities. Recently, although in vivo animal models have been considered as the benchmark for evaluating drug efficacy, it should be noted that mice, monkeys, and other animals are not humans and therefore can’t fully reflect the true physiological conditions in the human body [15, 95]. In fact, it has been demonstrated that translating the results obtained from these animal models into clinical application is a challenging task. The PDX model, which entails the engraftment of tumor tissue into immunocompromised mice, presents a disparity between the tumor microenvironment in mice and that in humans. Furthermore, the transplanted tumor tissue may undergo murine-like evolution, affecting the accuracy of PDX model of human tumor [96–98]. Additionally, these models are costly and time-consuming [99, 100]. The establishment of PDX models generally demands a time frame of around 4–8 months for preclinical applications, which can potentially result in missed therapeutic opportunities for cancer patients [101]. The establishment of PDX models typically necessitates around 4 to 8 months for preclinical applications. This duration may result in the potential missed opportunity for patients with cancer to receive optimal therapy in a timely manner [101, 102]. Additionally, in recent years, due to the rise of the animal protection movement, animal experiments are facing severe ethical challenges. PDOs serve as 3D tumor models that are cultured in vitro. These PDOs can be derived from tumors at various stages of development and necessitate only a minor fraction of tumor tissue for establishment and subsequent in vitro expansion [55, 63, 103–111]. In comparison, organoids, with their composition and structure more similar to primary tissues and ability to enable complex cellular interactions in a 3D environment, are better suited to address questions that cannot be resolved by in vivo models [112, 113]. Organoids offer several advantages for drug screening and other applications. Firstly, they exhibit a higher degree of similarity to cellular functions under physiological conditions, resulting in more stable biological transfection. Secondly, organoids possess relevant microenvironments and tissue structures, which may directly impact the drug sensitivity of tumor cells through cell-to-cell interactions [114]. Organoids faithfully replicate the architectural and functional characteristics of parental tissues, while also preserving histopathological features, genetic profiles, mutational landscapes, and even exhibiting responses to therapeutic interventions (Fig. 3) [115, 116].

Recently, ninety to ninety-five percent of drugs that have been proven safe in animal testing fail in human clinical trials due to the inability of animal testing to predict toxicity or lack of efficacy [117]. Repetitive studies of drugs that lack efficacy significantly prolong the drug development process, while unpredictable toxicity not only increases the duration of clinical trials but also brings about numerous unknown risks and side effects [118]. However, compared to traditional animal models, organoids offer significant advantages in simulating the genetic and epigenetic features of target tissues or organs, with short cycles, high throughput, and strong clinical relevance [119]. Tumor organoids exhibit three distinct advantages, namely, rapid cultivation speed, high screening throughput, and strong clinical relevance. This makes it possible to deeply explore the essence of human diseases and develop effective intervention measures, with broad application prospects in new drug development, drug sensitivity testing, exploration of new diagnostic and therapeutic strategies, discovery of new targets, understanding disease mechanisms, and regenerative medicine [64]. At the Shanghai Cancer Center of Fudan University, a total of 80 organoids were obtained from patients diagnosed with locally advanced rectal cancer who were undergoing neoadjuvant chemoradiotherapy. The organoids were subjected to in vitro chemoradiotherapy, revealing a noteworthy concordance between the chemoradiotherapy response of the organoids and that of the respective patients. The accuracy was determined to be 84.43%, with a sensitivity of 78.01%. The results also indicated a specificity of 91.97%, further highlighting the potential of patient-derived tumor organoids in predicting the response of tumor patients to chemotherapy, radiotherapy, and targeted therapy [120].

Despite the numerous advantages of organoids over other in vitro models, their use is still subject to limitations [121, 122]. For instance, organoids are non-vascularized and often lack some of the diverse cell types present in natural organs [123]. Most organoids are cultured in media supplemented with growth factors, which may interfere with functional/biochemical analyses and complicate cell collection and passaging [124, 125]. Moreover, the accumulation of growth factors around organoids may disrupt the natural morphological gradients of tissues. The presence of ECM also poses challenges for downstream applications.

**Table 2:** Comparison between Cell, PDOs and PDX models

Characteristics	Cell Models	PDO Models	PDX Models
Physiological manifestation	Limited models	Semi-physiological models	Physiological models
Establishment time	Short	Moderate	Long
Technical difficulty	Low	Low	High
Success rate	High	High	Low
Costs	Low	Moderate	High
Tissue size	Few biopsy	Few biopsy/surgical tissue	Numerous biopsy/surgical tissue
Vascularization	No	No	Yes
Immune system	No	No	Yes
Heterogeneity	No	Partial heterogeneity	Partial heterogeneity
Clinical significance	Low	High	High
Gene Editing	Easy	Easy	Difficult
Throughput screening	High	High	Low
Establishment of Biobank	Actionable	Actionable	Difficult to realize
Consistency	Low	High	High
Ex vivo, in vivo, or in vitro	Ex *vivo* or in vitro	Ex vivo, in vitro or in vivo	In vivo
Use of immunodeficient mice	NA	No	Yes

*PDO* patient-derived organoid, *PDX* patient-derived xenografts, *NA* not applicable

## Organoids for clinical application

**Fig. 4:**
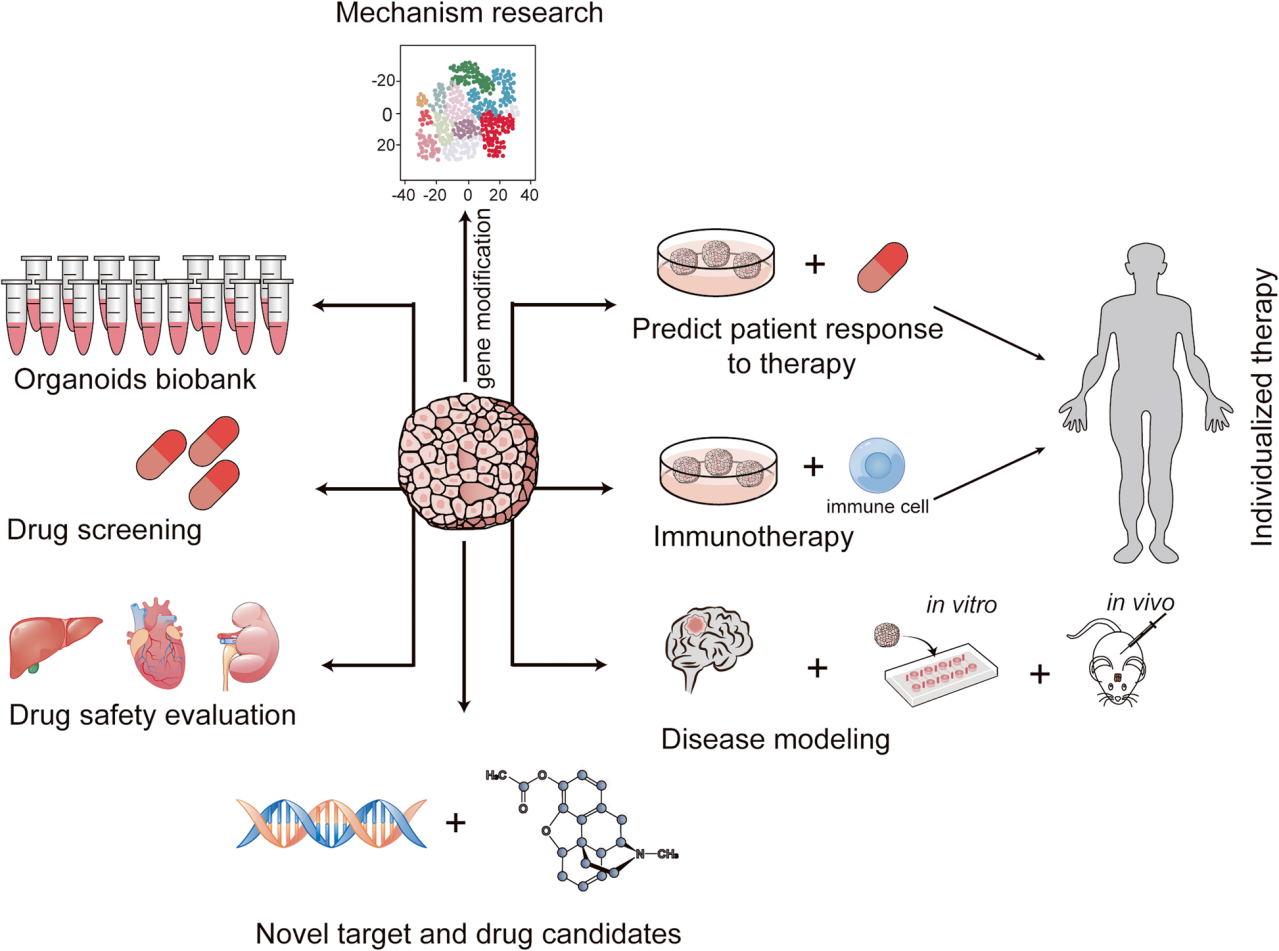
Organoid culture offers manifold potential applications, including emulation of pathological processes for disease modeling, organoid biobanking, high-throughput screening of anti-cancer drugs, evaluation of drug toxicity and side effects, CRISPR-Cas9 gene editing enabling fundamental research, identification of therapeutic targets and drug candidates, and customized therapy in clinical settings, predicting drug sensitivity

Currently, organoids have broad application prospects in organ development, precision medicine, regenerative medicine, drug screening, safety evaluation and other fields (Fig. 4) [77, 126, 127].

### Personalized therapy

Tumor organoids represent an emerging in vitro model that faithfully recapitulates the complexity of tumors in vivo [58, 126, 128–131]. Even after multiple generations of culture, tumor organoids successfully preserve the biological hallmarks of the primary tumor and exhibit robust genetic stability [78]. Tumor organoids have the ability to more accurately simulate the complex tumor environment and can be utilized for screening anti-tumor drugs, predicting patient responses to drug therapy, and guiding personalized treatment plans [132, 133].

Vlachogiannis et al. utilized cell cultures derived from rectal cancer patients to generate tumor organoids, conducted drug sensitivity tests, and subsequently administered the same chemotherapy to patients with rectal cancer [55]. Encouragingly, the results demonstrated that drugs found to be effective in the tumor organoids were successful in 88% of patients, while those that failed to demonstrate efficacy in the organoids also proved ineffective in 100% of patients [55]. These findings underscore the clinical relevance of tumor organoids in accurately predicting drug responses, thereby presenting the potential to guide individualized treatment strategies for patients afflicted with cancer [55]. Kim et al. employed an airway organoid culture system to successfully cultivate lung cancer organoids, demonstrating their utility in high-throughput drug screening and in vitro models to predict drug response in patients, potentially facilitating personalized cancer treatment strategies [134]. Tumor organoids, due to their multifunctionality, have been utilized in various immunotherapy methods. For instance, the use of patient-derived colon cancer organoids has led to a more profound comprehension of the therapeutic response of the bispecific antibody cibisatamab, which targets carcinoembryonic antigen (CEA) [135]. Wang Y conducted a preliminary investigation to examine the safety and antitumor efficacy of pyrotinib using tumoroids and corresponding xenografts derived from tumor samples obtained from patients with advanced lung adenocarcinoma characterized by the presence of the HER2-A775_G776YVMA insertion [136]. In contrast to afatinib, pyrotinib exhibits substantial growth inhibition of organoids and induces a notable reduction in tumor burden within PDX models [136]. Consistently, a phase II clinical trial involving 15 patients with HER2-mutant non-small cell lung cancer (NSCLC) demonstrated the therapeutic advantages of pyrotinib. The study revealed a notable objective response rate of 53.3% and a median progression-free survival of approximately 6.4 months among the treated patients [136]. In short, the advancements in tumor organoid technology hold significant promise in enhancing the efficacy and precision of in vitro drug response prediction. This, in turn, can furnish practical and objective treatment recommendations for patients.

### Organoid-based drug screening and identification of therapeutic targets

**Fig. 5:**
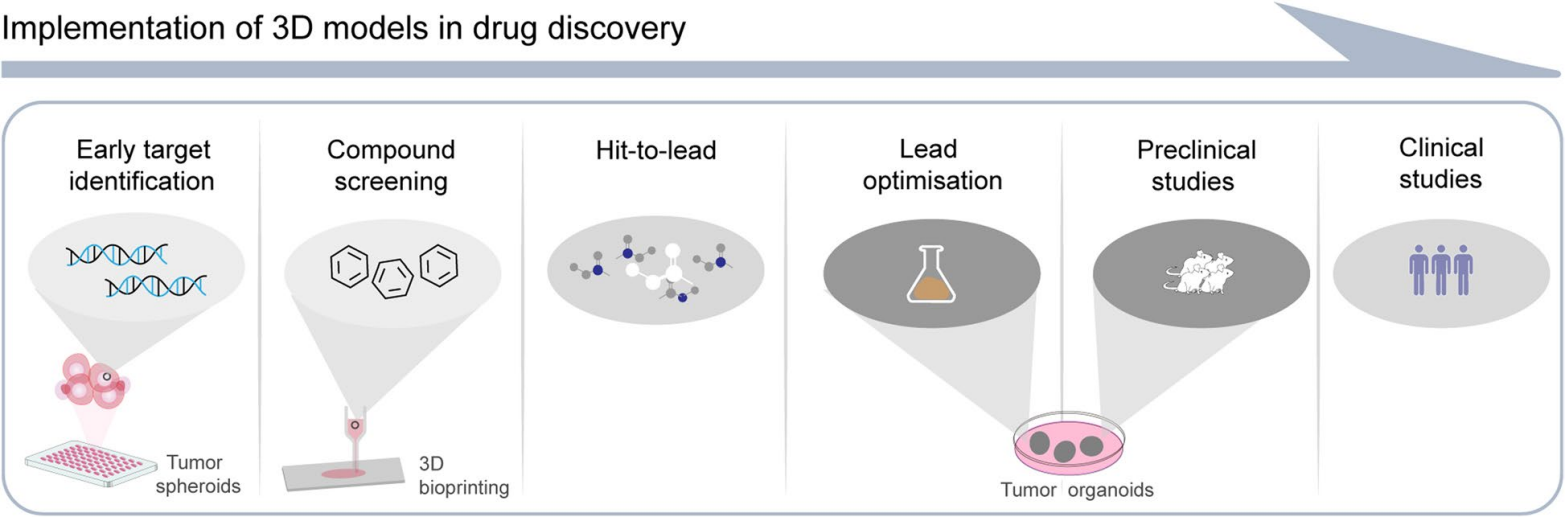
The drug discovery and development process can be broadly classified into six stages, comprising early target identification, compound screening, hit-to-lead, lead optimization, preclinical studies, and clinical studies. To hasten drug development, in vitro tumor models, such as tumor spheroids, tumor organoids, and 3D bioprinter models, have been instrumental. Tumor organoids, being more complex in vitro models, enable researchers to study tumor behavior in a more physiologically relevant environment. This can be especially beneficial when accurate in vivo recapitulation is critical

The process of drug discovery and development can be delineated into six overarching stages, namely: early target identification, compound screening, hit to lead, lead optimization, preclinical studies, and clinical studies (Fig. 5). The advancement of in vitro tumor models has greatly accelerated, resulting in the creation of various model designs such as tumor spheroids, tumor organoids, and 3D bioprinted models. Organoids, in particular, are employed not only to gain insights into potential therapeutic targets but also to expedite the development of innovative anti-tumor medications [137–139]. For instance, liver metastatic rectal cancer organoids have been utilized to study the effects of the SMAC mimetic LCL161 [139], while SCLC organoid lines have facilitated research on the novel CDK7 inhibitor YPN-005 [137]. Through an extensive high-throughput screening, focused on the interaction between patient-derived BCOs and tumor-specific cytotoxic T cells, researchers identified three noteworthy epigenetic inhibitors—BML-210, GSK-LSD1, and CUDC-101—which demonstrated significant antitumor effects [138]. Moreover, the inclusion of BML-210 exhibited a remarkable ability to enhance the sensitivity of BC to a PD-1 inhibitor [138]. By employing glioma organoids and human umbilical vein endothelial cells in a coculture system within a fibrin gel, the drug atorvastatin exhibited notable inhibitory effects on angiogenesis, which were found to be dose-dependent. Accompanying this effect was the downregulation of VEGF, CD31, and Bcl-2 [140]. This phenomenon suggests that atorvastatin holds promising potential as an agent for the treatment of cancer.

Regarding high-throughput drug screening, Driehuis [132] conducted a study evaluating 76 drugs across 30 PDOs obtained from pancreatic tumors. The significant finding from this investigation revealed the potential of the PRMT5 inhibitor EZP015556 as a drug capable of inhibiting MTAP-negative tumors. Furthermore, their comprehensive database of 30 pancreatic tumor organoids serves as a valuable platform for screening additional drug candidates. Kondo [141] presents an innovative system enabling high-throughput screening of 2,427 drugs, utilizing organoids derived from cancer tissue. The study specifically focuses on colorectal cancer organoids, which exhibit diverse sensitivities to these compounds. These findings lay a solid foundation for the development of personalized therapeutic approaches. Both the intrinsic tumor-suppressing or tumor-killing effects of drugs and the efficient delivery of drugs into tumors are crucial elements in cancer treatment. Using multicellular hepatocellular carcinoma (HCC) organoids, which incorporate HCC cells along with various stromal cells (endothelial cells, fibroblasts, and hepatic stellate cells), researchers demonstrated a strong association between the activation of Yes-associated protein/transcriptional coactivator with PDZ-binding motif signaling and stromal activation in HCC [142].

### Challenges and potential solutions in implementing organoids in clinical settings

Several challenging bottlenecks and difficulties persist within the field. First, the process of organoid establishment, maintenance, and passages remains costly. Second, there is notable variation in the success rates of establishing diverse cancer types in organoid models [143]. Third, a perfect replication of the full functionality of an original tumor remains a challenge for tumor organoids. Tumor tissue inherently possesses a highly heterogeneous and complex structure within the human body, while current organoids still fall short in fully capturing the heterogeneity exhibited by different types of tumors in vitro. While pluripotent stem cells have proven capable of generating various types of organ tissues that display a multitude of physiological functions, the inclusion of exogenous artificial ECM remains essential. Nevertheless, inconsistencies in mechanical properties of ECM, attributable to batch-to-batch variations and local changes, introduce disparities into organoid cultures. Consequently, such variability greatly restricts the clinical utilization and transplantation potential of organoids.

## Organoids for fundamental research

In addition to the aforementioned advancements in clinical applications, we present a comprehensive summary of the progress made by organoids in fundamental research, which, overall, still encounters certain technical limitations (Fig. 4).

### Human tumor model system

Standardized and high-quality biobank is an important carrier to preserve precious genetic resources. It is one of the most important links in basic and clinical research of major diseases, research and development of clinical diagnosis and treatment technology, drug research and development, and realization of precision medicine and translational medicine [144–148]. It is also a crucial link in original research of life sciences. Up to the present, an increasing amount of evidence has consistently supported the feasibility and significant advantages of utilizing lung cancer organoids (LCOs) [84, 149–156]. Kim M et al. successfully reported the establishment of organoids that effectively represent five distinct subtypes of LC [134]. During long-term in vitro passage, these LCOs demonstrated the ability to faithfully retain the morphological and histological characteristics, as well as the genomic variations, of the corresponding primary lung cancer [134]. Ganesh K et al. has curated a biobank containing 65 PDOs obtained from individuals diagnosed with primary, metastatic, or recurrent rectal cancer. They achieved an impressive 77% success rate across the entire dataset [108]. These tumoroids have been successfully generated using extremely small quantities of tumor tissues obtained from endoscopic biopsies [108]. Consistently, rectal cancer organoids adeptly recapitulate the molecular and histopathological features exhibited by the corresponding parental tumors [108]. Fadi Jacob et al. reported methods for generating and biobanking patient-derived GBM organoids, which establishes a rich resource for basic and translational GBM research [78]. Norman Sachs et al. constructed > 100 primary and metastatic breast cancer organoids [31]. 3D organoid constructs have also been employed to investigate the relationship between infectious pathogens and corresponding cancers. For instance, the presence of Helicobacter pylori in gastric cancer, Salmonella enteritidis in gallbladder cancer, various hepatitis viruses in liver-related cancers, herpes viruses in nasopharyngeal cancer, and other infectious agents demonstrate a strong association with the initiation and progression of cancer [157–161]. Through the implementation of co-culturing systems involving diverse pathogens, organoids prove to be exceptional models for investigating these diseases. To investigate the correlation between Salmonella typhi infection and gallbladder cancer, Scanu [50] fabricated mouse gallbladder organoids. The findings of this study revealed that enteric Salmonella typhi could induce malignant transformation in the aforementioned organoids, thereby confirming Salmonella Typhi as a pathogenic factor in the development of gallbladder cancer.

Pigs and monkeys are model animals that help explore human diseases and assess drug efficiency and toxicity, but high costs limit their use. Haonan Li et al. found that porcine colonic organoids were more similar to human colonic organoids in drug toxicity [162]. The establishment of colon organoids in pigs and monkeys can contribute to the mechanism of intestinal homeostasis as well as drug development and toxicity studies. Tumor organoid models are increasingly recognized as a convenient and versatile approach for studying various diseases, with a particular emphasis on human cancers [40, 107, 163]. Sylvia F Boj et al. successfully established organoid models derived from both normal and neoplastic murine and human pancreas tissues. These pancreatic organoids can be efficiently generated from resected tumors and biopsies, withstand cryopreservation, and manifest distinct ductal and disease-stage-specific characteristics [107]. Amanda Linkous et al. established a model system whereby we can retro-engineer patient-specific GBM using patient-derived glioma stem cells (GSCs) and human embryonic stem cell (hESC)-derived cerebral organoids [48]. Moreover, tumor research has also employed models such as tumor prognosis and tumor apoptosis models. In summary, organoids assume a crucial role in the field of tumor research, particularly in regards to modeling. Whether it involves delving into molecular mechanisms such as tumor induction and metastasis, or contributing to clinical practices like predicting therapeutic targets, organoids offer valuable assistance to researchers in gaining a deeper understanding of the mechanisms underlying tumor initiation, progression, and potential treatment strategies.

### Insightful investigation of disease mechanisms using PDOs

Tumoroids reliably maintain the critical characteristics of primary parent tumors, enabling the investigation of mechanisms associated with therapy resistance. Through the utilization of a patient-derived tumor organoid model for CRC, this study demonstrates that treatment with adipocyte exosomes can diminish the sensitivity of CRC cells to oxaliplatin. Furthermore, it validates that MTTP (microsomal triglyceride transfer protein) inhibits iron-induced cell death, thus facilitating the development of drug resistance [164]. In a separate study, it was discovered that aberrant cyclin P expression promoted stemness-like phenotypes in intestinal cancer organoids, frequently resulting in tumor recurrence, metastasis, and resistance to therapy [165]. The organoids have also broad application prospects in fundamental research and clinical diagnosis and treatment. Combined with CRISPR/ Cas9-mediated gene editing technology, biological functions of genes can be studied in organoids [166–172]. Common colon cancer gene mutation has been introduced into human intestinal stem cells in culture or organoids derived from normal intestinal epithelium, including *KRAS*, *APC*, *TP53*, *SMAD4*, and *PIK3CA* genes [53, 173]. Breast cancer organoids have been used to elucidate the pathways of tumor occurrence and metastasis [174]. For example, the roles of integrin and human epidermal growth factor receptor, FZD6 and Malat1 long non-coding RNAs in pretumor tissues have been confirmed, revealing the importance of RANK ligand and JNK in the development of breast cancer and breast cancer. Statistical analyses have revealed that as many as one in five tumors are linked to infections. Nevertheless, the precise mechanism underlying their occurrence within the human body remains unclear. Kyle W McCracken et al. reported the modelling human development and disease in pluripotent stem-cell-derived gastric organoids [175]. By employing human gastric organoids as a model to study the pathogenesis of human disease, researchers discovered that H. pylori infection rapidly led to the association of the virulence factor CagA with the c-Met receptor, triggering signaling activation and inducing epithelial proliferation [175].

### Applications in drug discovery and toxicology studies

Currently, the most commonly used models for anti-tumor drug (e.g., small molecule drugs, antibodies drugs and chemotherapy agents) screening and evaluation are the tumor cell-line model and the tumor tissue animal transplantation model, which have played a crucial role in the development of anti-tumor drugs and tumor treatment [176–178]. However, with the deepening of tumor research, it has become increasingly clear that these models are insufficient to address the challenges of tumor drug therapy [179]. They cannot accurately predict how well a drug will perform in the body. The tumor tissue animal transplantation model can retain tumor heterogeneity and simulate the tumor microenvironment to some extent, making it a valuable tool for tumor drug screening [30, 177, 180]. Nevertheless, it has several limitations, including a long experimental period, high cost, and inability to facilitate high-throughput screening. In contrast, the organoid model has the potential to significantly shorten the traditional drug development cycle.

Organ toxicity stands as a significant contributor to the failure and subsequent withdrawal of drugs during the development process, even after receiving approval. Conventional toxicological screening methods, employing cell lines and animal models, frequently prove inadequate in forecasting adverse reactions in humans. Organoids have emerged as a versatile tool for drug research and development, drug toxicity detection, high-throughput drug screening, pharmacokinetic research, and other fields [77, 181–187]. In terms of drug efficacy testing, Kita et al. [188] generated organoid models of human and mouse gastric cancer to simulate the typical features of human gastric cancer and the pathway of abnormal activation signal pathway change. Through drug screening of human gastric cancer organoids, they identified organoids with different responses to conventional chemotherapy drugs, including 5-fluorouracil (5-FU), irinotecan, epirubicin, oxaliplatin, and docetaxel. The successful interference of drugs with abnormal activation pathways in the model demonstrates the potential use of organoids as models for testing therapeutic response. In drug toxicity testing, normal organoids can be used to screen for drugs that specifically target tumor cells without harming healthy cells. Kostadinova et al. [189] constructed 3D rat and human liver organoids in vitro. After incubation with selected hepatotoxic drugs, the researchers indicated that the model was better at detecting drug-induced toxicity, including species-specific drug effects, compared with monolayer hepatocyte culture.

### Identification of novel cancer biomarkers

Organoids also play a significant role in the exploration and identification of novel tumor biomarkers [16, 190, 191]. Shoichi Ukai pioneered the establishment of gastric cancer organoids and successfully acquired 5-FU-resistant gastric cancer organoids [190]. They discovered that KHDRBS3 potentially plays a significant role in the development of acquired resistance in gastric cancer stem cells [190]. This finding highlights KHDRBS3 as a potential marker for predicting treatment outcomes and prognosis in patients with gastric cancer. G Emerens Wensink conducted a meta-analysis that encompassed 17 studies utilizing PODs in personalized tumor response tests since 2018 [192]. The main objective of this analysis was to assess the effectiveness of predicting tumor treatment response. The current findings present a promising outlook, indicating that utilizing PDOs for individualized tumor response testing holds clinical value as a predictive biomarker for cancer patients. If the establishment of PDOs for the majority of patients can be achieved within a practical timeframe, this potential predictive biomarker has the potential to advance personalized medicine for a specific group of patients who urgently require reliable predictive biomarkers. Rosemary Millen builds organoids from tumor tissues of head and neck cancer patients and exposes them to chemotherapy, radiotherapy and targeted drugs [193]. The results showed that there was a significant correlation between organoid sensitivity and clinical response. Organoid gene editing based on CRISPR-Cas9 has been applied to biomarker validation. They show how organoids can be used to explore biomarker potential in targeted therapeutic settings and determine that inhibition of PRMT5 may be an effective way to treat HNSCC.

### Advantages of organoids in fundamental research

Organoids bring many advantages to fundamental research. Firstly, the main advantage of organoids over standard cell culture methods is the 3D spatial arrangements of the cells in culture, which mimic their natural morphologies. Therefore, organoids preserve the cellular and molecular mechanisms of the cells in comparison to standard cell cultures or even spheroids. These properties make organoids excellent models to study organ generation and functions for whole organs as well as specific regions [64]. Secondly, organoids allow interactions among multiple cell types and resemble organ-like complex structures, which is impossible to achieve by any standard monolayer cultures, spheroids or even co-cultures of two or more cell types. Organoids provide an opportunity to study cell-to-ECM interactions as well as cell-to-cell interactions on a 3D level [65]. Thirdly, the use of human organoids eliminates species-specific mechanisms and offers effective alternatives to animal models to study human organs and hence provides an opportunity to perform mechanistic studies within the “human model” system.

Tumoroids, multiple organoids specific for individual organs or multiorgan-on-a-chip allow disease pathogenesis or collective treatment responses of an individual to be studied [116]. Fourthly, having more resemblance to in vivo systems compared to spheroids or standard cell cultures, organoids can be employed efficiently to model mechanisms of diseases and test the efficacy of drug candidates and other therapeutic interventions for diseases [116].

## Current challenges and future directions

Currently, organoid culture technology is experiencing a technological explosion and a surge in scientific research results. The industry’s growth potential is vast, yet it also confronts significant challenges.

Firstly, the organoid technology lacked standardized protocols for organoid construction and operation [194–196]. This has resulted in significant variability in the success rates of organoid generation across different cancer types, with reproducibility and consistency remaining major bottlenecks in organoid development [143, 197]. In organoid culture, excessive human involvement and insufficient automation can lead to significant errors caused by system randomness. Additionally, there are still gaps in the detection and evaluation of organoids, which will limit the efficient research and clinical translation of organoids [198]. For example, real-time monitoring of organoids using optical, electrochemical, and other methods is still relatively lacking. Secondly, the utilization of mouse-derived ECM substitutes, such as Matrigel or basement membrane extract, and in certain organoid cultures, the inclusion of fetal calf serum (used for WNT-conditioned medium production), introduces uncertain external factors that could potentially impact the results of experiments, including drug screening [82, 124, 199]. Notably, it has been demonstrated that the presence of serum adversely affects long-term growth [199]. The ongoing matter of enhancing organoid culture efficiency through further optimization of the culture medium remains a significant issue deserving of attention. Thirdly, most organoids lack inherent vascularized structures [200, 201]. Consequently, as organoid size increases, they are constrained by hypoxia and the accumulation of metabolic waste, which may ultimately culminate in tissue necrosis [201]. Constructing tumor vasculature in organoids is a necessary step in dissecting angiogenic signaling pathways and developing effective therapeutic strategies in the future study. Fourthly, organoids, although valuable in numerous respects, currently present limitations as they lack critical components, such as surrounding mesenchymal cells, immune cells, and stromal cells [202–205]. Accurately modeling the intricate immune microenvironment surrounding tumors proves to be a challenging endeavor. Fifthly, tissue samples prepared for organoid generation are only small parts of the whole tumor. The higher heterogeneity of tumors questions the reliability of substituting small pieces for whole tumor tissues. Sixthly, while medical ethics serve to safeguard the interests and dignity of research subjects, they can also impede the execution of clinical trials involving organoids.

In the future, the research direction of organoid technology will pay more attention to improving the biosimilarity and function of the model, and enhance its application value in clinical treatment and drug development. Specifically, tumor organoids can be co-cultured with non-tumor cells or induced pluripotent stem cell-derived organoids, compensating for the lack of non-tumor cell components within the tumor organoids. This enables the investigation of the interaction between tumor organoids and immune cells in the absence of an immune system. Organoid technology needs to more accurately simulate the physiological and pathological processes of human organs. Organoids can also be processed in large quantities, allowing for modification of their DNA sequences using genome editing techniques and subsequent analysis employing various cellular and molecular anatomical techniques. In addition, organoids can be integrated with advanced technologies such as 3D printing and organ chips to create engineered organs [206]. The utilization of microfluidic technology facilitates the formation of blood vessel structures and enables fluid communication, thus simulating complex circulatory systems. Last but not least, organoids have the potential to revolutionize the field of clinical science and drastically impact the way we approach diagnosis, treatment, and personalized medicine. ne significant impact of organoids on clinical practice lies in disease modeling. By culturing organoids from patient-derived cells, clinicians can simulate specific diseases in vitro, enabling them to better understand disease progression, identify potential therapeutic targets, and develop more effective treatments. Their ability to replicate human organ physiology in a laboratory setting offers unparalleled opportunities for advancing patient care, reducing treatment timelines, and increasing the overall efficiency of clinical practice.

## Conclusion

PDOs have emerged as an outstanding platform for both fundamental research and translational medicine. These organoids faithfully preserve the morphological characteristics, genomic profiles, and mutational landscapes of the parental tumors. In the realm of precision medicine, the integration of PDOs with clinical applications holds the potential to genuinely achieve precise treatments based on individualized patient differences. PDOs exhibit broad prospects in the realms of research and development, as well as screening of anti-tumor drugs and targeted therapies. PDOs substantially contribute to disease modeling, research on disease mechanisms, and drug screening advancements. This encompasses cancer modeling, enabling the study of tumorigenesis and cancer progression, along with predicting drug responses, optimizing treatments, and facilitating the discovery of novel patient-specific anti-tumor drugs. Nevertheless, the current organoids still exist certain limitations, necessitating further studies to standardize the organoid culture techniques and reduce associated costs. These advancements are crucial to enhance the integration of organoids with blood vessel, nerve, and immune components, ultimately resulting in the establishment of a comprehensive tumor microenvironment system.

## Data Availability

Not applicable.

## Abbreviations

GBM: glioblastoma multiforme
2D: two-dimensional
3D: three-dimensional
PDOs: patient-derived organoids
RBC: red blood cell
ECM: extracellular matrix
PDX: patient-derived xenograft
5-FU: 5-fluorouracil
CEA: carcinoembryonic antigen
HCC: hepatocellular carcinoma
hESC: human embryonic stem cell
GSCs: glioma stem cells
GLOBOCAN: Global Cancer Observatory
IARC: International Agency for Research on Cancer
iPSCs: induced pluripotent stem cells
WGS: whole-genome sequencing
CNV: Copy number variation
SNV: single nucleotide variant
CRC: colorectal cancer
NSCLC: non-small cell lung cancer
HCC: hepatocellular carcinoma
LCOs: lung cancer organoids
